# Hepatitis B virus modulates store-operated calcium entry to enhance viral replication in primary hepatocytes

**DOI:** 10.1371/journal.pone.0168328

**Published:** 2017-02-02

**Authors:** Jessica C. Casciano, Nicholas J. Duchemin, R. Jason Lamontagne, Laura F. Steel, Michael J. Bouchard

**Affiliations:** 1 Program in Molecular and Cellular Biology and Genetics, Graduate School of Biomedical Sciences and Professional Studies, Drexel University College of Medicine, Philadelphia, Pennsylvania, United States of America; 2 Program in Microbiology and Immunology, Graduate School of Biomedical Sciences and Professional Studies, Drexel University College of Medicine, Philadelphia, Pennsylvania, United States of America; 3 Department of Microbiology and Immunology, Drexel University College of Medicine, Philadelphia, Pennsylvania, United States of America; 4 Department of Biochemistry and Molecular Biology, Drexel University College of Medicine, Philadelphia, Pennsylvania, United States of America; Indiana University, UNITED STATES

## Abstract

Many viruses modulate calcium (Ca^2+^) signaling to create a cellular environment that is more permissive to viral replication, but for most viruses that regulate Ca^2+^ signaling, the mechanism underlying this regulation is not well understood. The hepatitis B virus (HBV) HBx protein modulates cytosolic Ca^2+^ levels to stimulate HBV replication in some liver cell lines. A chronic HBV infection is associated with life-threatening liver diseases, including hepatocellular carcinoma (HCC), and HBx modulation of cytosolic Ca^2+^ levels could have an important role in HBV pathogenesis. Whether HBx affects cytosolic Ca^2+^ in a normal hepatocyte, the natural site of an HBV infection, has not been addressed. Here, we report that HBx alters cytosolic Ca^2+^ signaling in cultured primary hepatocytes. We used single cell Ca^2+^ imaging of cultured primary rat hepatocytes to demonstrate that HBx elevates the cytosolic Ca^2+^ level in hepatocytes following an IP_3_-linked Ca^2+^ response; HBx effects were similar when expressed alone or in the context of replicating HBV. HBx elevation of the cytosolic Ca^2+^ level required extracellular Ca^2+^ influx and store-operated Ca^2+^ (SOC) entry and stimulated HBV replication in hepatocytes. We used both targeted RT-qPCR and transcriptome-wide RNAseq analyses to compare levels of SOC channel components and other Ca^2+^ signaling regulators in HBV-expressing and control hepatocytes and show that the transcript levels of these various proteins are not affected by HBV. We also show that HBx regulation of SOC-regulated Ca^2+^ accumulation is likely the consequence of HBV modulation of a SOC channel regulatory mechanism. In support of this, we link HBx enhancement of SOC-regulated Ca^2+^ accumulation to Ca^2+^ uptake by mitochondria and demonstrate that HBx stimulates mitochondrial Ca^2+^ uptake in primary hepatocytes. The results of our study may provide insights into viral mechanisms that affect Ca^2+^ signaling to regulate viral replication and virus-associated diseases.

## Introduction

Viruses are obligate intracellular parasites, and many viruses have developed methods to subvert host cell signal transduction pathways and factors to support their own replication and survival [1]. Ca^2+^ is a universal and versatile intracellular second messenger, and Ca^2+^ signaling affects almost every cellular process, ranging from fertilization to cell proliferation and cell death [2–5]. The amplitude, frequency, and spatiotemporal patterning of an intracellular Ca^2+^ signal can elicit differential modulation of Ca^2+^-binding proteins and Ca^2+^-dependent effectors, thereby regulating numerous cellular responses and functions [5, 6]. Not surprisingly, many pathogens, including a large number of DNA and RNA viruses, encode proteins that alter normal cellular Ca^2+^ signaling; for viruses, altered Ca^2+^ signaling typically enhances viral replication [6, 7]. Due to the versatile nature of a Ca^2+^ signal, modulation of intracellular Ca^2+^ signaling is an ideal mechanism for viruses to create a cellular environment that is permissive to viral replication. Although Ca^2+^ signaling is a common target of many viruses, for most of these viruses, the mechanisms that underlie viral-mediated regulation of Ca^2+^ levels and signals remain undefined.

Intracellular Ca^2+^ signaling is extremely dynamic and tightly controlled. The cytosolic Ca^2+^ level ([Ca^2+^]_c_) is normally maintained at approximately 100 nM, and subtle changes in [Ca^2+^]_c_ can have major effects within a cell [2, 3]. In non-excitable cells, such as hepatocytes, increases in [Ca^2+^]_c_ mainly stem from two sources: Ca^2+^ release through IP_3_ receptors (IP_3_R) on the endoplasmic reticulum (ER), the major intracellular Ca^2+^ store, and Ca^2+^ influx from outside the cell. Excess Ca^2+^ is removed from the cytosol by ATPases such as plasma membrane (PM) Ca^2+^-ATPases (PMCA), which pumps Ca^2+^ out of the cell and into the extracellular matrix, and sarcoplasmic reticulum Ca^2+^-ATPases (SERCA), which pump Ca^2+^ from the cytosol into the ER [2, 3, 8]. Deregulation of [Ca^2+^]_c_ and/or cytosolic Ca^2+^ signaling can affect development and progression of many diseases, including heart disease, schizophrenia, bipolar disorder, and Alzheimer’s disease. Moreover, disruption of Ca^2+^ signaling has been linked to cancer initiation, progression, metastasis, invasion, and tumor-associated angiogenesis [2, 5, 9]. Altered [Ca^2+^]_c_ has also been implicated in the proliferation of human hepatoma cells [10, 11], and modified expression of Ca^2+^ signaling regulators has been associated with the development of hepatocellular carcinoma (HCC) [12]. Interestingly, altered Ca^2+^ signaling and elevated [Ca^2+^]_c_ has been observed in cells with replicating hepatitis B virus (HBV) and hepatitis C virus (HCV); these viruses are currently the most common causes of HCC. These observations provide support for the notion that viral modulation of cellular Ca^2+^ signaling pathways could directly influence the development of viral-associated diseases.

Globally, approximately 350 million people are chronically infected with HBV. HBV infection is associated with life-threatening liver diseases, including cirrhosis and HCC [13–15]. HBV is a member of the *Hepadnaviridae* family, and its genome is a small, partially double-stranded DNA of 3.2 kb in length containing four overlapping open reading frames that encode just seven proteins [15]. The smallest HBV protein, HBx, is the main viral regulatory protein and stimulates viral replication both *in vitro* and *in vivo* [16–22]. HBx is localized mainly to the cytoplasm and nucleus of cells, with a small fraction present on the outer mitochondrial membrane (OMM), where HBx also interacts with the voltage-dependent anion channel (VDAC) [23–25]. HBx is thought to play a significant role in HBV pathogenesis, and many HBx activities, including its stimulation of viral replication and modulation of cell proliferation, apoptosis pathways, transcription, and other cell signaling pathways, are dependent on Ca^2+^ signaling [26].

The results of previously published studies have demonstrated that HBx expression causes an increase in the basal [Ca^2+^]_c_ in some established cell lines [27, 28] and that cytosolic Ca^2+^ signaling is required for several steps in HBV replication in these cells, including capsid assembly, activation of the HBV polymerase, and replication of the HBV genome [29–32]. While some studies have identified activities of the HBx protein that are Ca^2+^-dependent in normal hepatocytes [30, 31, 33], the natural site of an HBV infection, whether HBx directly elevates [Ca^2+^]_c_ in normal hepatocytes has not been assessed. We previously demonstrated that HBx enhances extracellular Ca^2+^ influx and mitochondrial Ca^2+^ uptake in human hepatoblastoma HepG2 cells [34]; however, HBx activities can have cell-specific consequences, necessitating a direct analysis of HBx effects on Ca^2+^ levels in normal hepatocytes [26, 35] For studies reported here, we have used cultured primary rat hepatocytes as a model system of normal hepatocytes; we previously showed that cultured primary rat hepatocytes can serve as a surrogate model for analyzing HBV effects in primary human hepatocytes [36, 37]. We now show that HBV, via HBx, increases the plateau [Ca^2+^]_c_ in cultured primary rat hepatocytes following induction of an IP_3_-linked Ca^2+^ response and that HBx elevation of [Ca^2+^]_c_ stimulates HBV replication in normal hepatocytes. We used both targeted and transcriptome-wide approaches to compare expression levels of store-operated Ca^2+^ (SOC) channel components and other Ca^2+^ signaling factors in HBV-expressing and control primary hepatocytes. HBV had no effect on the transcript level of any SOC channel component or Ca^2+^-signaling regulator that was detected in our analyses. Instead, we directly link HBx elevation of [Ca^2+^]_c_ and HBx stimulation of HBV replication in hepatocytes to increased Ca^2+^ entry into hepatocytes through mitochondrial regulation of SOC entry (SOCE). Cumulatively, the results of our studies suggest that HBV (and HBx) does not affect the expression level or activity of SOC channel components but does increase, and likely prolong, Ca^2+^ entry through SOC channels to stimulate HBV replication in normal hepatocytes. The results from this study contribute to our current understanding of mechanisms that regulate HBV replication in normal hepatocytes and HBx effects that could influence the development of HBV-associated HCC. These studies may also provide insights into mechanisms that other viruses might employ to alter [Ca^2+^]_c_ and Ca^2+^ signaling to enhance their own replication.

## Materials and methods

### Animal studies

Surgery and isolation of hepatocytes from rats, and this study, was approved by the Institutional Animal Care and Use Committee of Drexel University College of Medicine and complied with the Animal Welfare Act, the Policy on Humane Care and Use of Laboratory Animal, and the NIH Guide for the Care and Use of Laboratory Animals (2011).

### Isolation and maintenance of cultured primary rat hepatocytes

Hepatocytes were isolated from male Sprague-Dawley rats by a two-step perfusion method, as previously described [100]. Cells were plated on collagen-coated tissue culture plates or slides (see below) and maintained in Williams E medium supplemented with 2 mM L-glutamine, 1 mM sodium pyruvate, 4 μg/ml insulin-transferrin-selenium, 5 μg/ml hydrocortisone, and 5 ng/ml epidermal growth factor at 37°C in 5% CO_2_. Hepatocytes were monitored for maintenance of hepatocyte morphology and expression of hepatocyte-specific mRNAs (see below) throughout the time course of our experiments.

### Confirmation of differentiated hepatocytes

Levels of albumin, transferrin, and hepatocyte nuclear factor 4 alpha (HNF4α) mRNAs were monitored as markers of differentiated rat hepatocytes [38, 40]. RNA was isolated from rat hepatocytes immediately following isolation (0 hr) and following 48 hours of culture on collagen-coated glass coverslips (48 hr) using Trizol (Invitrogen), according to manufacturer’s protocols. RNA was treated with DNase and then converted to cDNA using M-MuLV reverse transcriptase (New England BioLabs, Inc). qPCR was performed with *Power* SYBR Green PCR master mix (Invitrogen), according to manufacturer’s instructions. Plasmids encoding gene portions for albumin, transferrin, and HNF4α (see below) were used to generate a standard curve for each target. These standard curves enabled us to convert Ct values to cDNA copy numbers, which we used in our comparisons. Levels of connexin 43, a marker present in other types of liver cells, including liver sinusoidal endothelial cells (LSECs), and in dedifferentiated hepatocytes, but not in differentiated hepatocytes [39], were also monitored; the absence of this marker in our hepatocytes confirmed both the purity of our preparation and the differentiated status of our cells. We also isolated RNA from primary rat LSECs and generated cDNA as described above. qPCR was performed on cDNA generated from LSECs and from hepatocytes at 0 hr and 48 hr for levels of connexin 43. A plasmid encoding a gene portion for connexin 43 (see below) was used to generate a standard curve, which enabled us to convert Ct values to cDNA copy numbers, which we used in our comparisons. Primers used for each target are listed in Table 1.

**Table 1:** Primer list.

Primer	Sequence		
Albumin-F	5'-	AAA GCA CTG GTC GCA GCT GTC CG	-3'
Albumin-R	5'-	TCG CTG GCT CAT ACG AGC TAC TGC C	-3'
HNF4a-F	5'-	AGT GCT GCC TTG GAC CCA GCC T	-3'
HNF4a-R	5'-	GGC ACA CAG GGC ACT GAC ACC C	-3'
Transferrin-F	5'-	TTA CGG GTG CCC CCA AGG ATG GAC T	-3'
Transferrin-R	5'-	ATT TCA CTG GCG CGC TGT CGA TGG	-3'
Connexin43 -F	5'-	GAG GTG CCC AGA CAT GGG T	-3'
Connexin43-R	5'-	AGC ACT GAC AGC CAC ACC T	-3'
Stim1-F	5'-	GCC ACA GCA TGG CCT GGG	-3'
Stim1-R	5'-	CCA GGA TTG TCT TCT TGG CC	-3'
Stim2-F	5'-	AAT GCT GCT CTT CGG GCT GT	-3'
Stim2-R	5'-	GCA TGG TGG ACT CAG AGA CAT	-3'
Orai1-F	5'-	GCC TGG TCT TTA TCG TCT TTG CCG	-3'
Orai1-R	5'-	CCT CTG TGG TCC ACG TGG TCC	-3'
Orai2-F	5'-	GGA TTA CCG AGA CTG GGT CC	-3'
Orai2-R	5'-	GGC TGA GGG TAC TGG TAC TTG	-3'
Orai3-F	5'-	TAC CTC GAC CTT ATG GGG GC	-3'
Orai3-R	5'-	TGC AGG CAC TAA ATG CCA CT	-3'
AldoB-F	5'-	AGG TGC CCC GCT TGC AGG AAC	-3'
AldoB-R	5'-	GCT GGC GTA GCG AGC CAG AGC	-3'
β-actin-F	5'-	TGG CGT AGC GAG CCA GAG C	-3'
β-actin-R	5'-	GGC CCA CGA TGG AGG GGA AG	-3'

### Plasmids and transfections

Plasmids encoding gene portions for albumin, transferrin, HNF4α, and connexin 43 were constructed as follows: a portion of each gene was PCR amplified and cloned into the pcDNA3.1- vector. Primary rat hepatocytes were transfected using Lipofectamine 2000 (Invitrogen) according to manufacturer’s instructions. Full-length HBx, containing N-terminally Flag-tagged HBx cloned into the pcDNA3.1(-) vector (pcDNAHBx), has been previously described [25]. pGEMHBV and pGEMHBV*7 have been previously described [41–43]. Briefly, pGEMHBV*7 is identical to pGEMHBV except that a point mutation in the codon for the seventh amino acid of HBx generates a stop codon, preventing HBx expression [42].

### Reagents

Fura-4F/acetoxymethyl ester (Fura-4F/AM), pluronic acid, and ionomycin were purchased from Invitrogen; ATP was purchased from Amresco; 2-aminoethyldiphenyl borate (APB) was purchased from Cayman Chemical; vasopressin (Vp) was purchased from Calbiochem; lanthanide (La^3+^) and sulfobromophthalein (BSP) were purchased from Sigma; and thapsigargin (TG) was purchased from Acros Organics.

### Antibodies

The anti-HBV core (HBcAg) antibody was purchased from Dako-Cytomation, the anti-ß actin antibody was purchased from Sigma, the anti-HBx antibody was purchased from ViroStat, Inc, and the antibodies for Stim1 and Stim2 were purchased from Cell Signaling.

### Single cell [Ca^2+^]_c_ measurements

Hepatocytes were plated on collagen-coated 15 mm glass coverslips immediately following isolation. 24 hours post plating, hepatocytes were transfected with 1 μg control vector (pGEM or pGEMHBV*7 or pcDNA3.1(-)) or 1 μg HBx expression vector (pGEMHBV or pcDNAHBx) and 0.2 μg dsRED. 24 hours post transfection, cells were washed twice with HEPES-buffered balanced salt solution (HBSS) (121 mM NaCl, 5 mM NaHCO_3_, 25 mM HEPES, 4.7 mM KCl, 1.2 mM MgSO_4_, 1.2 mM KPO_4_, 2 mM CaCl_2_, 10 mM glucose, 0.25% [w/v] bovine serum albumin [BSA], 200 μM BSP, pH 7.4). Hepatocytes were loaded with 5 μM Fura-4F-AM in HBSS supplemented with 0.02% pluronic acid for 40 min in 37°C shaker at 100 rpm, washed twice with HBSS, and then imaged using an Olympus 1X71 inverted microscope equipped with a 20X objective used for Fura imaging and a cooled charged-coupled-device camera. HBx-expressing and control cells were identified by dsRED expression and selected for [Ca^2+^]_c_ measurements. To initiate Ca^2+^ release from the ER, 100 μM ATP or 100 nM Vp was added to the imaging buffer. Cytosolic Ca^2+^ signals were monitored by alternately exciting the dye at 340 nm and 380 nm and collecting emission at 510 nm with MetaFluor fluorescence ratio imaging software (Molecular Devices, Downingtown, PA). Fura-4F fluorescence images were recorded every 2 seconds, and the change in [Ca^2+^]_c_ was calculated as the change in the Fura-4F 340/380 nm ratio. Following each experiment, auto-fluorescence was estimated by treating cells with 10 μM ionomycin and 20 mM MnCl_2_ in Ca^2+^-free ECM to quench the Fura signal. Any remaining signals at 340 or 380 nm were subtracted prior to calculating the 340/380 nm ratio.

### SOCE measurements

To image SOCE, hepatocytes were plated, transfected, and loaded with Fura-4F, as described above. Following loading, hepatocytes were washed and imaged in Ca^2+^-free HBSS. Cells were treated with 2 μM TG for 10 min to drain the ER calcium store and activate SOCE. 1 mM CaCl_2_, 2 mM MnCl_2_, or 2 mM BaCl_2_ was then added to the buffer and cells were imaged an additional 10 min.

### [Ca^2+^]_m_ measurements

To measure [Ca^2+^]_m_, hepatocytes were plated, transfected, and loaded with Fura-4F, as described above. Following loading, hepatocytes were washed and imaged in HBSS. Cells were stimulated with 100 μM ATP to induce mitochondrial Ca^2+^ uptake. Following 3 min, cells were treated with 10 μM CCCP to release mitochondrial Ca^2+^ into the cytosol and imaged for an additional 5 min.

### Statistical analysis

For all Ca^2+^ imaging studies, data are reported as population means ± standard error (SE). Statistical significance was determined using a two-tailed, nonparametric Student *t* test. A *P* value of ≤ 0.05 is considered statistically significant.

### RT-qPCR of SOC channel and mitochondrial Ca^2+^ signaling components

Primary rat hepatocytes were transfected with the vector control (pGEM) or the pGEMHBV expression plasmid. Cells were collected 24 hours post transfection and total RNA was isolated using Trizol (Invitrogen), according to manufacturer’s instructions. RNA was treated with DNase and then converted to cDNA using M-MuLV reverse transcriptase (New England BioLabs, Inc). qPCR was performed with *Power* SYBR Green master mix (Invitrogen), according to manufacturer’s instructions. Fold differences in the expression of SOC channel and mitochondrial Ca^2+^ signaling component mRNAs were calculated by the delta-delta Ct method [101]. Primers used for each target are listed in Table 1.

### Recombinant adenoviruses

Recombinant adenoviruses used in this study have been previously described [33, 37]. Briefly, recombinant adenoviruses that express GFP (AdGFP) or a greater-than unit length copy of the HBV genome (AdHBV) were used to infect cultured primary hepatocytes. All the recombinant adenoviruses express GFP, which was used to monitor infection efficiency and to ensure that 100% of hepatocytes were infected.

### HBV replication assay

Primary rat hepatocytes were infected with AdHBV and treated with indicated SOCE inhibitors for 48 hours. HBV replication was analyzed by Southern blot analysis as previously described [29].

### Northern blot analysis

Primary rat hepatocytes were infected with AdHBV and treated with indicated SOCE inhibitors for 48 hours. For control and APB-treated hepatocytes, total RNA was isolated using Trizol (Invitrogen) according to the manufacturer’s instructions. Poly(A) + RNA was isolated using oligo (dT)-cellulose columns (Molecular Research Center, Inc.) according to the manufacturer’s instructions, followed by northern blot analysis as previously described [86]. For control and La^3+^-treated hepatocytes, total RNA was isolated using the Ambion mirVana isolation kit (Life technologies) according to the manufacturers instructions. 7μg of total RNA was glyoxalated for 1 hour at 55°C, after which samples were separated for 1 hour on a 1% 0.01M NaPO_4_ agarose gel with vigorous buffer recirculation. The gel was transferred to a GENESCREEN membrane (Perkin Elmer) using 10X SSC overnight. The membrane was briefly rinsed in 2X SSC, then pre-hybridized according to the GENESCREEN membrane manufacturer’s instructions. An HBV- specific probe was generated using the Takara Random Primer DNA Labeling kit (Clonetech) and an HBV-genome length DNA fragment generated from digestion of the pGEM-HBV plasmid with the restriction enzyme AATII. Hybridization was carried out according to the GENESCREEN membrane manufacturer’s instructions. The membrane was washed 3 times in 2X SSPE/0.1% SDS, and then imaged using a Storm PhosphorImager.

### Primary rat hepatocyte transcriptome analysis

Rat transcriptome experiments have been previously described [55], and associated data are available as a GEO SuperSeries using accession number GSE68113.

## Results

### HBV increases [Ca^2+^]_c_ in cultured primary rat hepatocytes

We previously demonstrated that cultured primary rat hepatocytes, which are readily available, can serve as a surrogate model for studying HBV effects in primary human hepatocytes, which are not always available in large quantities. We have also reported that the HBV HBx protein has identical activities in cultured primary human and rat hepatocytes, providing further support for the use of primary rat hepatocytes in our studies [36, 37].

**Fig 1:**
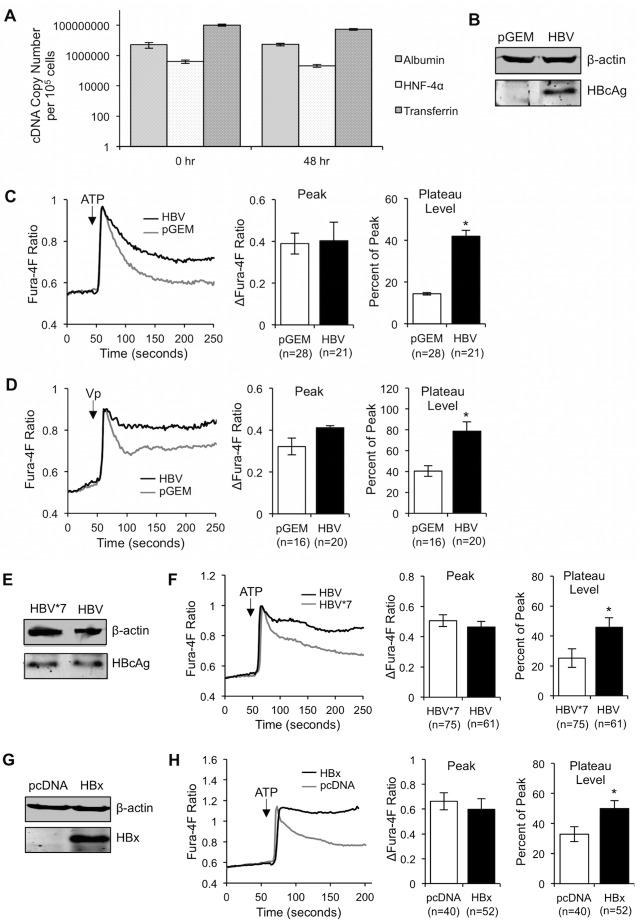
HBV/HBx increases [Ca^2+^]_c_ in primary rat hepatocytes. (A) RT-qPCR was performed on freshly isolated hepatocytes (0 hr) and on hepatocytes which had been plated for 48 hours (48 hr) for the hepatocyte-specific markers albumin, HNF4α, and transferrin. (B, C, and D) Primary rat hepatocytes were transfected with a control (pGEM) or HBV-expressing vector. 24 hrs post transfection, HBcAg expression was confirmed via western blot (B), and control and HBV-transfected cells were loaded with 5 μM Fura-4F and stimulated with 100 μM ATP (C) or 100 nM Vasopressin (Vp) (D). (E and F) Primary rat hepatocytes were transfected with an HBV-expressing or HBV(HBx-deficient) (HBV*7)-expressing vector. 24 hrs post transfection, equal levels of HBcAg expression were confirmed via western blot (E), and cells were loaded with 5 μM Fura-4F and stimulated with 100 μM ATP (F). (G and H) Primary rat hepatocytes were transfected with a control (pcDNA) or HBx-expressing vector. 24 hrs post transfection, HBx expression was confirmed via western blot (G), and cells were loaded with 5 μM Fura-4F and stimulated with 100 μM ATP (H). For all Ca^2+^-imaging experiments, the peak [Ca^2+^]_c_ was calculated as the change in the peak Fura-4F ratio and the basal Fura-4F ratio (R_peak_−R_basal_). The plateau [Ca^2+^]_c_ was calculated as a percentage of the peak [(R_plateau_−R_basal_) / (R_peak_−R_basal_)]. The data represent the means ± SE and are taken from at least three experiments from different hepatocyte preparations. *P < 0.05

To validate the differentiated status of our cultured primary rat hepatocytes, we first confirmed that our cultured primary rat hepatocytes express markers of differentiation throughout the time course of our experiments. Reverse transcription-quantitative PCR (RT-qPCR) was performed to quantitate levels of the hepatocyte-specific markers albumin, transferrin, and hepatocyte nuclear factor 4 alpha (HNF4α) [38–40]. These markers were expressed at high levels in hepatocytes immediately following isolation and also in hepatocytes that were maintained in culture for 48 hours, the longest time frame for our experiments (Fig 1A). We also monitored levels of connexin 43, a protein that is expressed in liver sinusoid endothelial cells, Kupffer cells, stellate cells, and in dedifferentiated hepatocytes, but not in differentiated hepatocytes [39]. We were unable to detect connexin 43 in our hepatocytes (data not shown), confirming both the purity of the hepatocyte preparation and the differentiation status of our cells. While some level of dedifferentiation is both normal and expected, the results of our studies indicate that within the timeframe of our experiments, our cells retained a high level of differentiation.

We first assessed whether HBV alters [Ca^2+^]_c_ in cultured primary rat hepatocytes. Intracellular Ca^2+^-signaling studies are typically performed by the addition of a Ca^2+^-inducing agonist and subsequent measurement of the resultant intracellular Ca^2+^ response in cells that are loaded with a Ca^2+^ indicator; we utilized this technique to compare the intracellular Ca^2+^ responses of control and HBV-expressing hepatocytes. HBV has a very narrow host range and can only naturally infect primary human hepatocytes [15]; we therefore delivered a copy of the HBV genome into cultured primary rat hepatocytes by transfection of a plasmid encoding a greater-than full length HBV genome (pGEMHBV). Previous studies have demonstrated that HBV that is expressed from this plasmid recapitulates all aspects of the HBV life cycle, with the exception of viral cell-entry steps [41–43]. Cultured primary rat hepatocytes were transfected with pGEMHBV or the vector control (pGEM); we assessed expression of HBV core protein (HBcAg) as a marker of general HBV protein expression (Fig 1B). HBV-expressing and control hepatocytes were loaded with the ratiometric fluorescent cytosolic Ca^2+^ indicator Fura-4F. The excitation wavelength of Fura-4F shifts from 380 nm to 340 nm upon Ca^2+^ binding; by calculating the Fura-4F 340/380 ratio, changes in the [Ca^2+^]_c_ in a single cell can be measured. We utilized ATP, a P2Y purinergic receptor agonist, to induce an IP_3_-linked cytosolic Ca^2+^ response in HBV-expressing and control hepatocytes (Fig 1C) [44–46]. ATP stimulation results in a biphasic Ca^2+^ response: the first phase, the release phase, reflects the initial release of Ca^2+^ from the ER and is represented by the initial peak in the Fura-4F ratio following ATP stimulation; the second phase, the recovery phase, occurs when the cell clears the excess Ca^2+^ from the cytosol at the same time that Ca^2+^ enters from outside the cell to refill the ER Ca^2+^ store, resulting in an elevated level of cytosolic Ca^2+^, referred to as the plateau [47]. When we compared this biphasic response between HBV-expressing and control hepatocytes, we observed no change in the initial Ca^2+^ peak following ATP stimulation. There was, however, a significant increase in the plateau Ca^2+^ level in HBV-expressing hepatocytes compared to control hepatocytes, indicating that HBV elevates [Ca^2+^]_c_ following stimulation of Ca^2+^ release from the ER. To confirm that this result was not specific to activation of the P2Y receptor, we also exposed hepatocytes to vasopressin (Vp), which binds to the vasopressin receptor V_1_ and induces an IP_3_-linked cytosolic Ca^2+^ response [48]. When Fura-4F-loaded HBV-expressing and control hepatocytes were stimulated with Vp, there was no change in the initial Ca^2+^ peak, but there was a significant increase in the plateau Ca^2+^ level (Fig 1D), similar to results observed when we treated HBV-expressing hepatocytes with ATP.

### HBV increases [Ca^2+^]_c_ via HBx

Many viruses modulate Ca^2+^ signaling via a viral regulatory protein; we and others have linked HBV regulation of cytosolic Ca^2+^ in some established cell lines to expression of the HBx protein [27, 29, 34, 49]. To determine if HBx has a similar effect in normal hepatocytes, we compared the Ca^2+^ response in primary rat hepatocytes expressing either wildtype HBV or a mutant HBV that does not express HBx (HBV*7). Equal expression levels of HBcAg in cells expressing HBV and HBV*7 was confirmed via western blot analysis (Fig 1E). When we compared the Ca^2+^ response to ATP in HBV- and HBV*7-expressing cells, we observed no change in the peak Ca^2+^ level but did observe a significant increase in the plateau Ca^2+^ level in HBV-expressing cells as compared to HBV*7-expressing cells (Fig 1F), indicating that HBx does, indeed, mediate HBV modulation of [Ca^2+^]_c_. We confirmed this result using primary rat hepatocytes that were transfected with an HBx expression plasmid compared to hepatocytes expressing the vector control (pcDNA); similar HBx regulation of [Ca^2+^]_c_ was observed when HBx was expressed alone or in the context of the full HBV genome (Fig 1H). HBx expression was confirmed by western blot analysis (Fig 1G).

### The HBV/HBx-mediated increase in [Ca^2+^]_c_ requires extracellular Ca^2+^ influx

**Fig 2:**
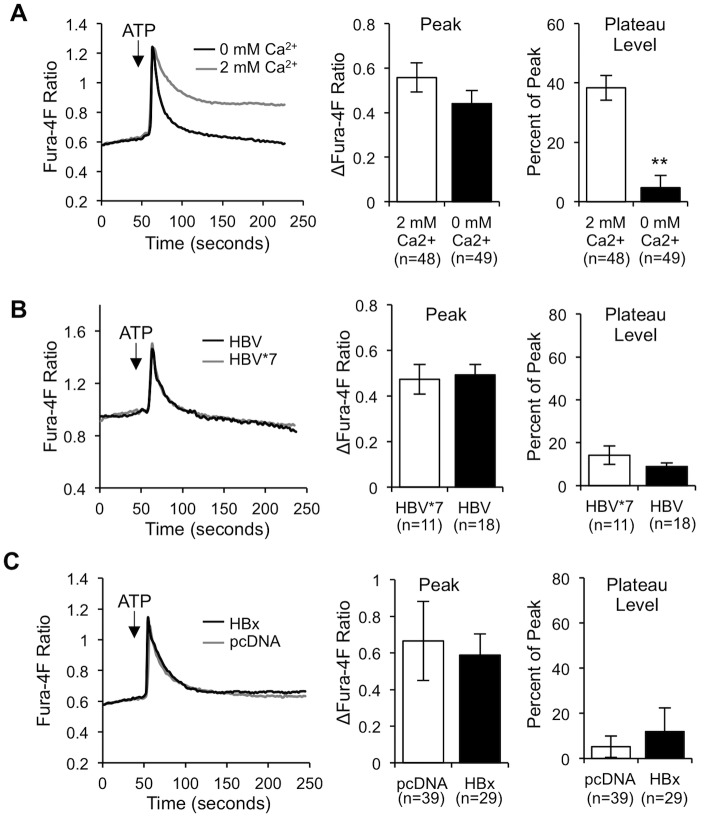
HBV/HBx requires extracellular Ca^2+^ influx to elevate [Ca^2+^]_c_. (A) Primary rat hepatocytes were loaded with 5 μM Fura-4F and stimulated with 100 μM ATP in Ca^2+^-free buffer (0 mM Ca^2+^) or in Ca^2+^-containing buffer (2 mM Ca^2+^). (B and C) Control and HBx-expressing primary rat hepatocytes were loaded with 5 μM Fura-4F and stimulated with 100 μM ATP in Ca^2+^-free buffer (0 mM Ca^2+^). Calculations were performed as described for Fig 1. The data represent the means ± SE and are taken from at least three experiments from different hepatocyte preparations. **P < 0.01

We next sought to define the mechanism underlying HBx elevation of the plateau Ca^2+^ level. It is well established that Ca^2+^ influx from outside the cell is responsible for the formation of the Ca^2+^ plateau following IP_3_-linked Ca^2+^ release from the ER [47, 50]. Indeed, when we stimulated control primary rat hepatocytes with ATP in Ca^2+^-free buffer, there was an extremely diminished Ca^2+^ plateau as compared to primary rat hepatocytes stimulated with ATP in Ca^2+^-containing buffer (Fig 2A). To determine whether Ca^2+^ influx mechanisms are involved in HBV elevation of [Ca^2+^]_c_, we treated Fura-4F-loaded HBx-expressing and control primary rat hepatocytes with ATP in Ca^2+^-free buffer, thus preventing any Ca^2+^ influx from outside the cell. Interestingly, the resulting plateau Ca^2+^ level in HBx-expressing primary rat hepatocytes was the same as in control primary rat hepatocytes (Fig 2B and 2C), indicating that HBV, via HBx, modulates Ca^2+^ influx mechanisms to elevate [Ca^2+^]_c_.

### HBV/HBx increases SOCE

The major mechanism of Ca^2+^ entry into hepatocytes is through SOC channels [51]. These channels consist of the ER transmembrane Stim proteins, Stim1 and 2, and the PM transmembrane Orai proteins, Orai1, 2, and 3. The Stim proteins contain an EF hand Ca^2+^-binding motif on the ER lumen side; upon depletion of the ER Ca^2+^ store, Ca^2+^ is no longer bound to this motif, resulting in aggregation of Stim near Orai, the pore-forming unit of the SOC channel. Stim binding to Orai causes the SOC channel to open, enabling Ca^2+^ to flow into the cell to refill the ER Ca^2+^ store [51, 52].

**Fig 3:**
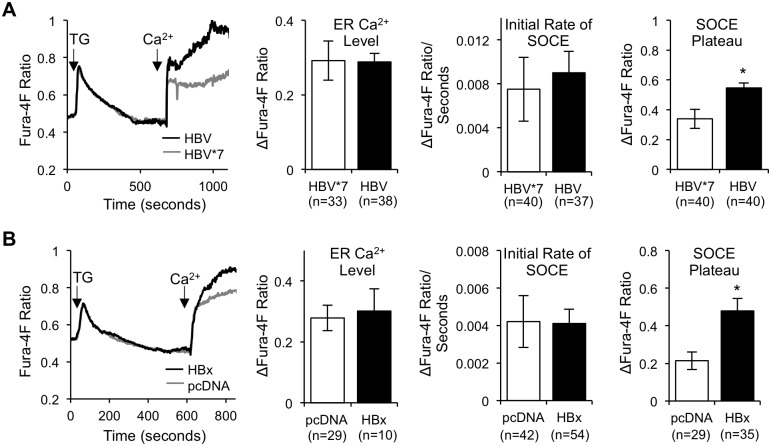
HBV/HBx stimulates SOCE. (A and B) Control and HBx-expressing primary rat hepatocytes were loaded with 5 μM Fura-4F and treated with 2 μM TG in Ca^2+^-free buffer for 10 min. 1 mM CaCl_2_ (Ca^2+^) was then added back to the imaging buffer, and Fura-4F ratios were recorded during the following 10 min. The ER Ca^2+^ level was calculated as the difference in the peak Fura-4F ratio following TG treatment and the basal Fura-4F ratio (R_TG peak_−R_basal_). The initial rate of SOCE was calculated as the slope representing Ca^2+^ influx during the initial 15 seconds following Ca^2+^ addition. The SOCE plateau was calculated as the difference in the plateau Fura-4F ratio following Ca^2+^ addition and the baseline Fura-4F ratio prior to Ca^2+^ addition (R_plateau_−R_baseline_). The data represent the means ± SE and are taken from at least three experiments from different hepatocyte preparations. *P < 0.05

To determined if HBV altered Ca^2+^ entry through SOC channels to regulate [Ca^2+^]_c_, we utilized a commonly employed method for activating SOCE and subsequently measuring the flow of Ca^2+^ through the SOC channel. Fura-4F-loaded HBx-expressing and control primary rat hepatocytes were imaged in Ca^2+^-free buffer and treated with the SERCA inhibitor, thapsigargin (TG). The ER is naturally leaky, and the addition of TG causes a complete depletion of the ER Ca^2+^ store, thereby activating the SOC channel. Following ER store depletion, Ca^2+^ was added back to the imaging buffer; increased Fura-4F ratios reflect Ca^2+^ entering the cell through the open SOC channel [53]. When we compared the peak Ca^2+^ level following TG treatment, indicative of the ER Ca^2+^ level, and the initial rate of SOCE, we saw no change between HBx-expressing and control primary rat hepatocytes (Fig 3), suggesting that HBx does not affect the level of Ca^2+^ in the ER nor the rate of Ca^2+^ entering through the SOC channel. When we compared the SOCE plateau, however, there was a significant increase in the level of Ca^2+^ in HBx-expressing primary rat hepatocytes, as compared to control hepatocytes, indicating that HBx expression, both on its own and in the presence of other HBV proteins, does cause more SOCE-regulated Ca^2+^ accumulation in the cytosol of primary rat hepatocytes (Fig 3).

### SOCE is required for HBV replication

**Fig 4:**
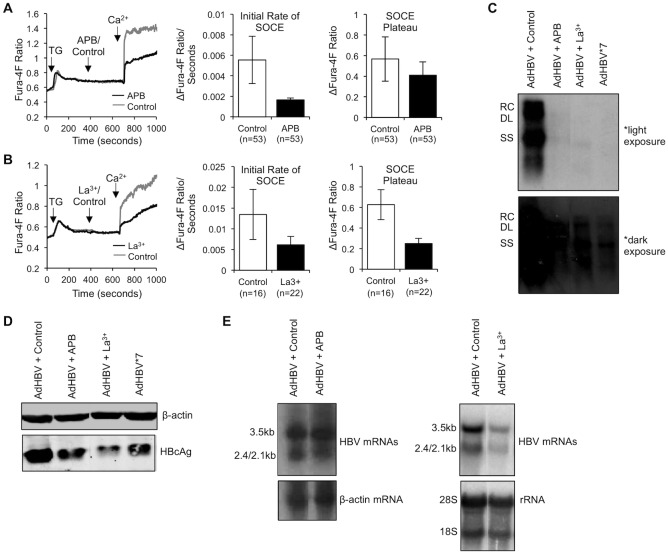
SOCE is required for HBV replication. (A and B) Primary rat hepatocytes were loaded with 5 μM Fura-4F and treated with 2 μM TG in Ca^2+^-free buffer, followed by treatment with DMSO or the SOCE inhibitor 50 μM APB (A) or 100 nM La^3+^ (B) before the addition of 1 mM CaCl_2_ (Ca^2+^). Calculations were performed as described for Fig 3. The data represent the means ± SE and are taken from at least three experiments from different hepatocyte preparations. (C-E) Primary rat hepatocytes were infected with a recombinant adenovirus expressing HBV (AdHBV) and treated with the SOCE inhibitors for 48 hrs or infected with a recombinant adenovirus expressing an HBx-deficient HBV (AdHBV*7). Levels of HBV replication (Southern blot) (C), HBV core protein expression (western blot) (D), and HBV mRNAs (northern blot) (E) were assessed.

Previous studies have shown that Ca^2+^ signaling is required for HBV replication in various cell types [29, 30, 34]; therefore, we next tested whether SOCE was specifically required to stimulate HBV replication in normal hepatocytes. We infected primary rat hepatocytes with a recombinant adenovirus encoding HBV (AdHBV). We previously reported that to facilitate measurement of HBV replication in primary hepatocytes, a high percentage of the hepatocytes must express HBV [25]. Under optimal transfection conditions, we have observed that only 30–40% of hepatocytes are transfected; however, AdHBV infection is extremely efficient and can deliver the HBV genome to close to 100% of the cultured hepatocytes. We utilized two SOCE inhibitors, 2-aminoethoxydiphenyl (APB) and lanthanide (La^3+^), to assess the requirement for SOCE in HBV replication (Fig 4A and 4B) [47]. Upon AdHBV infection, primary rat hepatocytes were treated with either 50 μM APB or 1 μM La^3+^ for 48 hours, and HBV replication was then measured via Southern blot analysis as previously described [29]. Due to the partially double-stranded nature of the HBV genome, HBV replication appears as a smear with three distinct bands that represent three replicative intermediates: relaxed circular (RC), double-stranded linear (DL), and single-stranded linear (SS). Interestingly, treatment with both SOCE inhibitors significantly decreased levels of HBV replication to a similar level of replication that occurs with HBx-deficient HBV (HBV*7) (Fig 4C). We also assessed the effect of SOCE inhibition on expression levels of HBV core protein (Fig 4D) and HBV mRNAs (Fig 4E) in order to pinpoint the specific stage of HBV replication that SOCE influences. We found that while both APB and La^3+^ treatment resulted in a reduction in HBV core expression, detected via western blot analysis, only treatment with La^3+^ resulted in reduced levels of HBV mRNAs, detected via northern blot analysis. The significance of decreased HBV core expression and a reduction in HBV mRNA expression in the presence of SOCE inhibition is not entirely clear but suggests that HBx regulation of SOCE could be affecting expression of HBV mRNAs or proteins. Cumulatively, these results demonstrate that HBx affects HBV replication in primary rat hepatocytes by modulating Ca^2+^ influx through SOC channels to elevate [Ca^2+^]_c_.

### HBV does not alter levels of SOC channel components

**Fig 5:**
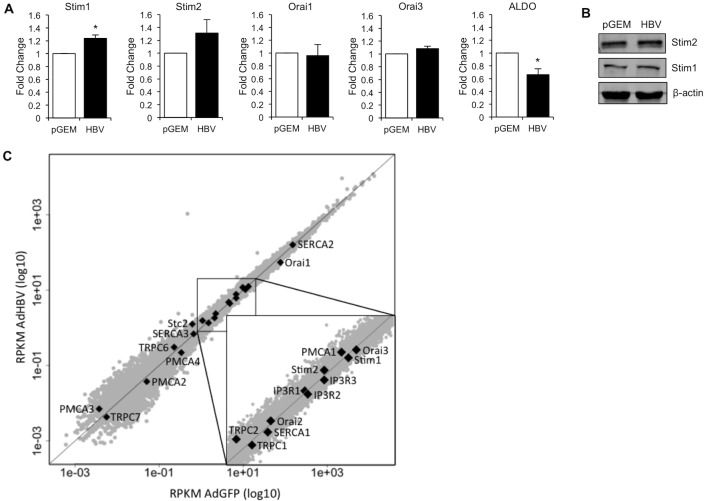
HBV does not alter levels of Ca^2+^ signaling-related genes. (A) Primary rat hepatocytes were transfected with a control (pGEM) or HBV-expressing vector. 24 hrs post transfection, RNA was isolated and RT-qPCR was performed for expression of Stim1 and 2, Orai1 and 3, ALDO B, and Actin mRNAs. (B) Primary rat hepatocytes were transfected with a control (pGEM) or HBV-expressing vector. 24 hrs post transfection, hepatocytes were collected and western blot analysis was performed to analyze Stim1 and 2 and ß actin protein levels. (C) Primary rat hepatocytes were infected with AdGFP or AdHBV. 24 hrs post infection, total RNA was isolated and RNA-seq analysis was performed. Expressed genes were plotted on a log10 scale (grey). Ca^2+^ signaling-related genes were highlighted (black dots) to show that they do not deviate from the expected slope of 1 (black diagonal line), which would represent no difference in gene expression between AdGFP- and AdHBV-infected hepatocytes. Points along the middle of the plot were expanded (inset) for clearer representation.

To further define the effect of HBV on SOCE, we monitored expression levels of SOC channel components in HBV-expressing and control primary rat hepatocytes. HBV regulates the expression of many proteins by activating transcription [22, 26], and studies have indicated that altered expression levels of Stim and Orai proteins can impact SOCE [51, 54]. We used RT-qPCR to assess the level of Stim1 and 2 and Orai1 and 3 mRNA transcripts in HBV-expressing and control primary rat hepatocytes (Fig 5A). Expression levels of Orai2 are extremely low in hepatocytes [54] and difficult to detect via RT-qPCR, and we therefore did not include Orai2 in this analysis. We previously showed that the levels of ALDO mRNA are decreased in the presence of HBV [36], and we included this target as a control for our assay. We observed equal Stim2, Orai1, and Orai3 mRNA expression levels in HBV-expressing and control cells and a slight increase in Stim1 mRNA (~1.2 fold). Although this slight increase in Stim1 mRNA was statistically significant, whether this increase is biologically relevant is unclear, as we did not detect an increase in Stim1 protein levels within the time frame of our studies (Fig 5B). It was not possible to measure protein expression for all SOC channel components because antibodies for detecting these components in rat cells are not available.

We confirmed our RT-qPCR results and expanded our analysis to other factors that control Ca^2+^ signals in hepatocytes with data generated from an unbiased sequencing of the poly-A-selected RNA transcriptome (RNA-seq) of RNA isolated from primary rat hepatocytes that were infected with AdGFP or AdHBV (Fig 5C and Table 2). This analysis is described in detail in [55] and was conducted on two independent isolations of primary rat hepatocytes, referred to in Table 2 as Datasets I and II. Using this data, we confirmed our targeted RT-qPCR results by comparing levels of transcripts that code for the canonical SOC channel components and also investigated whether HBV alters levels of transcripts that code for other proteins that could influence SOCE and cytosolic Ca^2+^ signaling. We detected mRNA transcripts for Stim1 and 2 and Orai 1, 2, and 3 and, consistent with our qPCR results and with the findings of others, we observed equal mRNA expression of Stim1 and 2 in control hepatocytes, whereas Orai2 was expressed at much lower levels in control hepatocytes than Orai1 or 3 [54] (Table 2). Importantly, when we compared mRNA expression of these factors between control and HBV-expressing primary rat hepatocytes, we observed no change in expression, supporting our conclusion that HBV does not alter expression of SOC channel components to enhance SOCE (Fig 5C and Table 2). In addition to the canonical components of the SOC channel, members of the transient receptor potential cation (TRPC) channel family can bind to Stim or Orai proteins to facilitate Ca^2+^ influx into the cell [51, 56–59]. We detected TRPC1, 2, 6, and 7 mRNAs in control hepatocytes; our detection of expression and expression levels of specific members of the TRPC family in hepatocytes is consistent with previously reported observations [60, 61]. Importantly, when we compared expression levels of the detected members of the TRPC family in control and HBV-expressing primary rat hepatocytes, we detected no change (Fig 5C and Table 2), suggesting that HBV does not regulate mRNA transcript levels of canonical or noncanonical components of the SOC channel. We also detected PMCA-1, 2, 3, and 4 mRNAs and SERCA-1, 2, and 3 mRNAs. Interestingly, PMCA1 and SERCA2 mRNAs seem to have the highest expression levels in primary rat hepatocytes, as compared to PMCA-2, 3, and 4 or SERCA-1 and 3, respectively (Table 2) [62]; although it is important to note that mRNA levels might not directly correlate with protein levels [63]. HBV expression did not alter the levels of PMCA-1, 2, 3, or 4 mRNAs or SERCA-1, 2, or 3 mRNAs (Fig 5C and Table 2), suggesting that HBV does not regulate mechanisms that pump Ca^2+^ out of the cytoplasm, at least at the mRNA transcript level. Finally, we also detected mRNA transcripts that code for IP_3_R-1, 2, and 3 (Table 2). In Dataset 1, transcript levels for all three isoforms appear to be expressed at equivalent levels; however, in Dataset 2, transcript levels for IP_3_R-3 are expressed at much lower levels than IP_3_R-1 or IP_3_R-2, which is consistent with previous reports (Table 2) [64]. It is unclear why the expression levels of the IP_3_Rs are so different in the two datasets, as similar differences in expression levels between datasets were not observed for the other factors that were analyzed. Importantly, HBV expression did not alter levels of the mRNAs coding for any isoform of IP_3_R (Fig 5C and Table 2), supporting our finding that HBV does not affect the peak Ca^2+^ level released from the ER following an IP_3_-linked Ca^2+^ response (Fig 1C).

**Table 2:** Ca^2+^ signaling-related gene expression in control and HBV-expressing primary rat hepatocytes.

			Dataset 1		Dataset 2		
Protein	Gene	Ensembl Gene ID	AdGFP RPKM	AdHBV RPKM	AdGFP RPKM	AdHBV RPKM	Avg Fold Change
Stim1	Stim1	ENSRNOG00000020425	11.279	10.334	9.152	9.579	0.981
Stim2	Stim2	ENSRNOG00000002956	6.807	7.740	8.042	8.867	1.120
Orai1	Orai1	ENSRNOG00000001336	77.046	54.370	36.921	25.958	0.704
Orai2	Orai2	ENSRNOG00000001427	2.223	2.372	0.236	0.354	1.283
Orai3	Orai3	ENSRNOG00000039730	13.320	12.443	12.672	12.583	0.964
TRPC1	Trpc1	ENSRNOG00000009601	1.498	1.350	1.390	1.303	0.919
TRPC2	Trpc2	ENSRNOG00000020188	1.078	1.549	1.661	2.857	1.579
TRPC3	Trpc3	ENSRNOG00000016070	-	-	-	-	N/A
TRPC4	Trpc4	ENSRNOG00000011133	-	-	-	-	N/A
TRPC5	Trpc5	ENSRNOG00000027233	-	-	-	-	N/A
TRPC6	Trpc6	ENSRNOG00000006324	0.228	0.306	0.136	0.171	1.296
TRPC7	Trpc7	ENSRNOG00000012727	0.006	0.004	0.000	0.000	0.382
PMCA1	Atp2b1	ENSRNOG00000004026	9.767	11.775	5.609	7.064	1.233
PMCA2	Atp2b2	ENSRNOG00000030269	0.051	0.038	0.041	0.060	1.102
PMCA3	Atp2b3	ENSRNOG00000017798	0.004	0.007	0.003	0.002	1.203
PMCA4	Atp2b4	ENSRNOG00000003031	0.338	0.220	0.071	0.075	0.852
SERCA1	Atp2a1	ENSRNOG00000047124	2.087	1.827	0.898	0.849	0.910
SERCA2	Atp2a2	ENSRNOG00000001285	151.273	158.998	99.776	105.235	1.053
SERCA3	Atp2a3	ENSRNOG00000017912	0.661	0.687	0.199	0.236	1.112
IP3R-1	Itpr1	ENSRNOG00000007104	4.532	4.775	3.798	3.758	1.021
IP3R-2	Itpr2	ENSRNOG00000001804	4.822	4.422	4.449	3.860	0.892
IP3R-3	Itpr3	ENSRNOG00000026651	6.810	6.158	0.630	0.763	1.057

While no change in gene expression does not necessarily correlate to no change in the protein level, HBV regulates protein expression primarily at the level of transcription, and the results of our total transcriptome analyses and RT-qPCR studies suggest that HBV does not regulate [Ca^2+^]_c_ and SOCE by altering expression levels of the Ca^2+^ regulators assessed here.

### HBV/HBx alters a secondary regulatory mechanism to enhance SOCE

In addition to alterations in the expression level of SOC channel components, several mechanisms have been proposed to regulate SOCE. High [Ca^2+^]_c_ in the vicinity of the SOC channel opening can initiate negative feedback signals that shut down SOCE; many SOCE regulatory mechanisms act by either augmenting or delaying negative feedback signals, resulting in dampened or enhanced SOCE, respectively [51, 65]. Thus far, we have shown that HBV expression enhances SOCE-regulated Ca^2+^ accumulation in the cytosol without altering the rate of SOCE (Fig 3). We have also shown that this enhanced Ca^2+^ accumulation is not due to altered levels of SOC channel components (Fig 5 and Table 2). These findings led us to hypothesize that HBV does not have a direct impact on the activity of the SOC channel but instead enables prolonged SOCE and enhanced cytosolic Ca^2+^ accumulation by affecting negative feedback mechanisms that would normally prevent further Ca^2+^ entry through the SOC channel.

**Fig 6:**
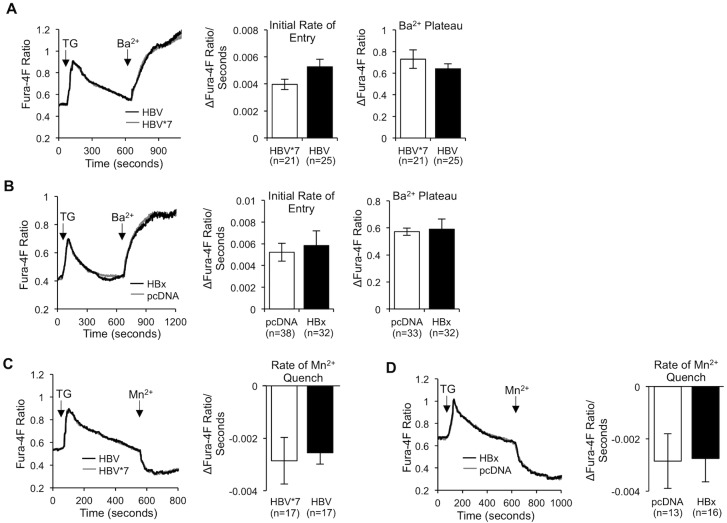
HBV/HBx does not directly affect SOC channel activity. (A, B, C, and D) Control and HBx-expressing primary rat hepatocytes were loaded with 5 μM Fura-4F and treated with 2 μM TG in Ca^2+^-free buffer for 10 min. 2 mM BaCl_2_ (Ba^2+^) (A and B) or 2 mM MnCl_2_ (Mn^2+^) (C and D) was then added back to the imaging buffer and ratios were recorded during the following 10 min. (A and B) The initial rate of Ba^2+^ entry was calculated as the slope representing Ba^2+^ influx during the initial 15 seconds following Ba^2+^ addition. The Ba^2+^ plateau was calculated as the difference in the plateau Fura-4F ratio following Ba^2+^ addition and the baseline Fura-4F ratio prior to Ba^2+^ addition (R_plateau_−R_baseline_). (C and D) The rate of Mn^2+^ quench was calculated as the slope representing Mn^2+^ influx during the initial 15 seconds following Mn^2+^ addition. (A, B, C, and D) The data represent the means ± SE and are taken from at least three experiments from different hepatocyte preparations.

To begin to test this hypothesis, we first used two additional methods to measure changes in SOCE. In addition to Ca^2+^, both barium (Ba^2+^) and manganese (Mn^2+^) can selectively enter through the SOC channel and bind Fura-4F. Fura-4F binding to Ba^2+^ results in a similar Fura excitation wavelength shift as when Ca^2+^ binds; however, when Fura-4F binds Mn^2+^, this results in quenching of the Fura signal [53]. Importantly, neither Ba^2+^ nor Mn^2+^ are handled within the cell in the same way as Ca^2+^, and neither ion stimulates negative feedback mechanisms that turn off the influx signal. Consequently, the use of Ba^2+^ or Mn^2+^ allows us to directly measure the activity of the SOC channel without influence from SOC channel regulatory mechanisms [66]. When we treated primary rat hepatocytes with TG to activate the SOC channel and then added Ba^2+^ or Mn^2+^ to the medium, we observed no change in the rate of Ba^2+^ entry or in the rate of the Mn^2+^ quench between HBx-expressing and control primary rat hepatocytes (Fig 6), confirming that HBV, via HBx, does not affect the rate (and likely the activity) of the SOC channel. When we analyzed the Ba^2+^ plateau following addition of Ba^2+^ however, we observed that the increase that we had observed when Ca^2+^ was added back was now lost in HBx-expressing cells (Fig 6A and 6B). Because Ba^2+^ entry is not impacted by SOCE regulatory mechanisms, this result led us to conclude that a SOC channel regulatory mechanism is likely responsible for facilitating increased SOCE in HBx-expressing cells. It is not possible to calculate the plateau formed by Mn^2+^ addition because Mn^2+^ binding to Fura-4F quenches the Fura signal. In all, these results indicate that HBx, both when expressed on its own and in the context of the entire HBV genome, likely does not directly influence the activity of the SOC channel, but perhaps alters SOC channel regulatory mechanisms, such that negative feedback signals that would normally shut down SOCE are not activated, resulting in prolonged SOC channel activation and, subsequently, increased Ca^2+^ accumulation in the cytosol.

### HBV/HBx enhances [Ca^2+^]_m_ to elevate SOCE

One SOCE regulatory mechanism that may be altered by HBV is mitochondrial Ca^2+^ uptake. Mitochondrial Ca^2+^ uptake is stimulated when there is a high local Ca^2+^ concentration in the vicinity of mitochondria, often the result of ER Ca^2+^ release or SOCE. By taking up this Ca^2+^, mitochondria can buffer negative feedback mechanisms and prolong a Ca^2+^ response [65, 67–71]. Altered or enhanced mitochondrial Ca^2+^ uptake during SOCE could result in increased Ca^2+^ accumulation in the cytosol. Interestingly, we previously reported that the mitochondrial membrane potential is increased in HBx-expressing primary rat hepatocytes [25], which is usually a precursor to increased mitochondrial Ca^2+^ uptake. Additionally, we also reported that mitochondrial Ca^2+^ levels ([Ca^2+^]_m_) were increased in HBx-expressing HepG2 cells [34]. These observations provide strong evidence that increased mitochondrial Ca^2+^ uptake likely occurs in HBV-expressing primary hepatocytes, which could potentially result in enhanced SOCE.

**Fig 7:**
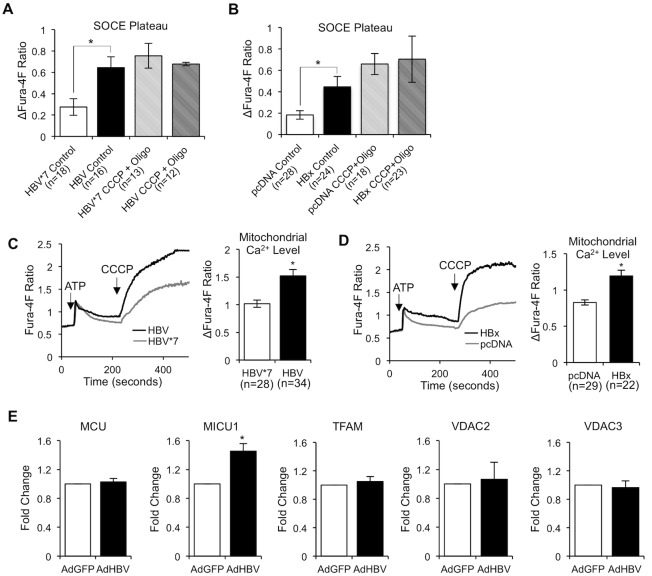
HBV/HBx enhances mitochondrial Ca^2+^ uptake to elevate SOCE. (A and B) Control and HBx-expressing primary rat hepatocytes were loaded with 5 μM Fura-4F and treated with 2 μM TG in Ca^2+^-free buffer, followed by treatment with DMSO or 10 μM CCCP and 5 mg/ml Oligomycin (Oligo) before the addition of 1 mM CaCl_2_ (Ca^2+^). Calculations were performed as described for Fig 3 (C and D) Control and HBx-expressing primary rat hepatocytes were loaded with 5 μM Fura-4F and treated with 100 μM, followed by 10 μM CCCP. The mitochondrial Ca^2+^level was calculated as the difference in the plateau Fura-4F ratio following CCCP addition and the baseline Fura-4F ratio prior to CCCP addition (R_plateau_−R_baseline_). (E) Primary rat hepatocytes were infected with a control (AdGFP) or HBV-expressing recombinant adenovirus (AdHBV). 24 hours post infection, RNA was isolated and RT-qPCR was performed for expression of MCU, MICU1, TFAM, VDAC2, and VDAC3 mRNAs. (A-E) The data represent the means ± SE and are taken from at least three experiments from different hepatocyte preparations. *P < 0.05

To first determine whether HBV does require mitochondrial Ca^2+^ uptake to enhance SOCE, we stimulated SOCE in HBx-expressing and control primary rat hepatocytes by exposing them to TG and simultaneously preventing mitochondrial Ca^2+^ uptake with the addition of the uncoupler carbonyl cyanide *m*-chlorophenyl hydrazine (CCCP), which disrupts the inner mitochondrial membrane potential, and oligomycin, an inhibitor of ATP synthase. Interestingly, when we prevented mitochondrial Ca^2+^ uptake, HBx-expressing cells no longer displayed enhanced SOCE (Fig 7A and 7B), indicating that mitochondria do play a critical role in mediating HBx regulation of SOCE. We next sought to determine whether HBx affects the [Ca^2+^]_m_ in primary hepatocytes. We first stimulated mitochondrial Ca^2+^ uptake in HBx-expressing and control cells with ATP treatment and then released [Ca^2+^]_m_ by the addition of CCCP. We observed an increase in [Ca^2+^]_m_ in HBx-expressing cells compared to control cells (Fig 7C and 7D). These results suggest that HBx stimulation of SOCE likely requires mitochondrial Ca^2+^ uptake and that HBx increases [Ca^2+^]_m_ following IP_3_R activation.

Taken together, these results support our hypothesis that HBx stimulates mitochondrial Ca^2+^ uptake during Ca^2+^ release from the ER and/or Ca^2+^ entry through the SOC channel. This mitochondrial uptake of Ca^2+^ dampens Ca^2+^-mediated inhibition of further Ca^2+^ release from the ER and/or Ca^2+^ entry through the SOC channel, thereby prolonging Ca^2+^ entry into the cytosol to elevate [Ca^2+^]_c_.

Lastly, in an effort to identify potential mechanisms underlying HBV modulation of [Ca^2+^]_m_, we performed an initial, targeted screen of mitochondrial Ca^2+^ regulators. Recent work has identified key regulators of mitochondrial Ca^2+^ signaling; alterations in the expression levels of these components has been shown to regulate mitochondrial Ca^2+^ uptake/efflux mechanisms, as well as [Ca^2+^]_m_ [72–76]. In order to determine whether expression levels of some key mitochondrial Ca^2+^ regulators are altered in HBV-expressing primary rat hepatocytes, we monitored mRNA levels of the mitochondrial Ca^2+^ uniporter (MCU), mitochondrial Ca^2+^ uptake 1 (MICU1), VDAC2, VDAC3, and mitochondrial transcription factor A (TFAM) in AdHBV-infected primary rat hepatocytes. MCU is a highly selective Ca^2+^ uniporter that transports Ca^2+^ across the inner mitochondrial membrane (IMM); MCU activity is regulated by several proteins, including MICU1, a single-pass, transmembrane protein located within the IMM. MICU1 contains a pair of Ca^2+^-binding EF-hand domains and functions as a Ca^2+^-sensing regulatory subunit of MCU [75–77]. VDAC proteins are a component of the mitochondrial permeability transition pore (MPTP), which can regulate mitochondrial Ca^2+^ uptake and efflux [78, 79]. Interestingly, a fraction of HBx interacts with VDAC3 on the OMM [24]. TFAM is essential for mitochondrial DNA transcription and replication; while TFAM primarily regulates transcription of mitochondrial genes, recent evidence suggests that TFAM can also regulate expression of nuclear genes, including SERCA [80]. Altered expression levels of these various components could impact mitochondrial Ca^2+^ accumulation. Interestingly, we observed equal MCU, VDAC2, VDAC3, and TFAM mRNA expression levels in HBV-expressing and control hepatocytes and a 1.5-fold increase in MICU1 expression (Fig 7E). These results suggest that HBV likely elevates expression of MICU1; however, whether this increase directly relates to the HBx-mediated elevation of [Ca^2+^]_m_ has yet to be determined. Additionally, these four factors are not the only regulators of mitochondrial Ca^2+^, and further studies will need to be performed to fully characterize HBx regulation of mitochondrial Ca^2+^.

## Discussion

Ca^2+^ is a tightly controlled and very dynamic universal second messenger. Changes in [Ca^2+^]_c_ can initiate signaling cascades that impact major cellular processes ranging from gene expression to cell growth and cell death [2–5]. Not surprisingly, it has been suggested that many human diseases may be caused by the remodeling or disruption of Ca^2+^ signaling pathways, resulting in inappropriate Ca^2+^ responses that are either too high or too low [12, 81]. Chronic infection with HBV is associated with various liver diseases and is the most common cause of HCC [13–15]. We have demonstrated that the HBV regulatory protein, HBx, alters normal cytosolic Ca^2+^ signaling in cultured, primary rat hepatocytes, and that HBx modulation of cytosolic Ca^2+^ is required for HBV replication.

HBx is the only regulatory protein encoded in the HBV genome; its expression stimulates HBV replication and is thought to influence the development of HBV-associated HCC [16–22, 26, 82–85]. We and others have shown that many HBx effects can be linked to HBx regulation of cytosolic Ca^2+^-dependent signal transduction pathways [28–31, 33, 49, 86–88]. Previous studies that have analyzed the direct effect of HBx on cytosolic Ca^2+^ levels were conducted in established cell lines, and most previous studies only assessed the effect of HBx that was expressed outside the context of the HBV genome [27, 28, 34, 87]. While these studies have provided valuable information regarding HBx effects in specific experimental contexts, it has become increasingly apparent that HBx effects can be context specific [26, 35]. In contrast, for the studies reported here, we used primary hepatocytes, the natural site of an HBV infection. We directly demonstrated that HBx elevates cytosolic Ca^2+^ levels in primary rat hepatocytes; this HBx effect was present when HBx was expressed alone or in the context of the HBV genome. Specifically, HBx altered a normal IP_3_-linked Ca^2+^ response, resulting in higher [Ca^2+^]_c_ than in control hepatocytes (Fig 1B–1H). The HBx-induced elevation of cytosolic Ca^2+^ required influx of extracellular Ca^2+^ (Fig 2B and 2C) through SOC channels (Fig 3). Importantly, SOCE-dependent HBx elevation of Ca^2+^ was essential for HBV replication in normal hepatocytes (Fig 4C), thus highlighting the significance of our studies.

Modulation of Ca^2+^ signaling may also be a key event in the pathogenesis of many other viruses, including hepatitis C virus (HCV), human immunodeficiency virus (HIV), human T-lymphotropic virus-1 (HTLV-1), rotavirus, influenza A virus, enterovirus, and human herpesviruses (HHV) [6, 7]. Because changes in Ca^2+^ levels and Ca^2+^ signaling can stimulate a wide range of cellular effects, viral modulation of Ca^2+^ is an ideal mechanism to create a cellular environment that is permissive to viral replication. Many viruses increase [Ca^2+^]_c_, similar to what we report here for HBV. Virus-mediated elevation of [Ca^2+^]_c_ is often linked to activities of a viral regulatory protein, although for many viruses, the mechanisms that underlie regulation of [Ca^2+^]_c_ are not fully defined [6, 7]. The components and nature of the SOC channel have only recently been defined, and so only a handful of viruses have been shown to modify this process, including HBV, rotavirus, and enterovirus [34, 89, 90]. Interestingly, rotavirus has been shown to activate SOCE via increased permeability of the ER Ca^2+^ store and a resultant decrease in ER Ca^2+^ levels. This increased ER permeability was linked to the rotavirus regulatory protein NSP4, which contains a viroporin activity [90, 91]. Alternately, modulation of SOCE by enterovirus was linked to activities of the viral regulatory protein LMP-1, which increased expression of Orai1 [89]. Importantly, we did not see a change in ER Ca^2+^ levels and HBx does not exhibit viroporin characteristics [92]. We also did not observe altered expression levels of SOC channel components, indicating that the method of SOCE modulation by HBV is different from that of either rotavirus or enterovirus. The results of this study could provide insights into mechanisms underlying viral regulation of [Ca^2+^]_c_ for other viruses that do not contain viroporins and that do not alter protein expression of SOC channel components.

We linked HBx elevation of cytosolic Ca^2+^ in normal hepatocytes to altered feedback mechanisms that negatively regulate SOCE, specifically mitochondrial regulation of SOC channels. By blocking mitochondrial uptake of Ca^2+^, we prevented HBx from elevating cytosolic Ca^2+^ levels that were associated with activation of SOCE (Fig 7A and 7B). HBx localizes to the OMM and interacts with the OMM protein VDAC [23–25]. Although not yet proven, the enhanced mitochondrial Ca^2+^ uptake seen in HBV- and HBx-expressing hepatocytes could be the result of HBx OMM localization and/or interaction with VDAC. Mitochondria normally take up cytosolic Ca^2+^ when the cytosolic Ca^2+^ level reaches 1 μM, which is 10 times the normal cytosolic concentration [76, 93, 94]. In HBV-infected hepatocytes, however, mitochondria may either have a lower threshold for taking up Ca^2+^, a larger capacity for Ca^2+^ storage, or are more sensitive or susceptible to taking up cytosolic Ca^2+^. When there is an IP_3_-linked Ca^2+^ response, mitochondria localized to the ER and/or SOC channels take up some of the released Ca^2+^, which dampens Ca^2+^-mediated Ca^2+^ inhibition of further Ca^2+^ release from the ER and/or Ca^2+^ entry through the SOC channel. This results in prolonged release of Ca^2+^ from the ER or prolonged entry of Ca^2+^ through SOC channels, causing a greater Ca^2+^ accumulation in the cytosol [65, 67–71]. Furthermore, we observed an HBV-mediated increase in MICU1 expression (Fig 7E) and elevated MICU1 could contribute to enhanced [Ca^2+^]_m_ in HBV-expressing hepatocytes. Our observed link of HBx elevation of cytosolic Ca^2+^ to mitochondrial-dependent processes is contradictory to a previous study conducted in HeLa and HepG2 cells where HBx elevation of cytosolic Ca^2+^ was associated with altered PMCA activity [87]. PMCA pumps excess [Ca^2+^]_c_ out of the cell; when PMCA activity is altered or impaired, [Ca^2+^]_c_ could accumulate in the cytosol [95, 96]. The authors of this previous study proposed that HBx elevation of cytosolic Ca^2+^ was directly caused by stimulating the pro-apoptotic activation of caspase 3, which subsequently cleaved and inactivated PMCA, decreasing Ca^2+^ efflux from the cytosol to the extracellular environment, resulting in elevated [Ca^2+^]_c_ [87]. It is important to note, however, that these studies were only conducted in a small number of HepG2 cells and HeLa cells with over-expressed HBx; the results were not confirmed in the context of the full HBV genome [87]. Moreover, cleavage of PMCA was only demonstrated in HeLa cells, which are derived from a cervical carcinoma. In contrast, we have demonstrated that HBx is normally anti-apoptotic in primary hepatocytes and does not activate caspase 3 [33, 36]. Overall, these contrasting results highlight the importance of directly assessing HBx activities in the context of the full HBV genome and in biologically relevant models systems such as cultured primary hepatocytes. Further defining the mechanism underlying HBV regulation mitochondrial Ca^2+^ uptake is the focus of our ongoing studies.

In addition to regulating HBV replication, HBx elevation of cytosolic Ca^2+^ may also be linked to the development, progression, and/or maintenance of HBV-associated diseases. Hepatocytes are constantly exposed to various insults, toxins, and growth hormones that could initiate an IP_3_-linked Ca^2+^ response [97–99]. In normal, non-HBV-infected hepatocytes, the resultant transient increase in [Ca^2+^]_c_ is quickly corrected by shuttling this excess cytoplasmic Ca^2+^ out of the cell, through PMCA, or back into the ER, through SERCA. In HBV-infected hepatocytes, however, this regulation of Ca^2+^ levels and signals is altered, and HBV-infected hepatocytes are subjected to a more substantial and persistent increase in cytosolic Ca^2+^. Persistent elevation of cytosolic Ca^2+^ likely stimulates downstream Ca^2+^-dependent effector proteins and signaling pathways to create a cellular environment that is permissive to HBV replication, but consequently alters hepatocyte physiology, potentially influencing the development of HBV-associated diseases.

In summary, we have shown for the first time that HBV, via HBx, modulates SOCE to elevate [Ca^2+^]_c_ in normal hepatocytes to stimulate HBV replication; we have linked this HBx effect to mitochondrial regulation of [Ca^2+^]_c_. HBx is the only HBV regulatory protein and is required for HBV replication in human hepatocytes [16–22]. HBx is also thought to play a major role in HBV pathogenesis [26]. HBV-related HCC develops after a decades-long HBV infection, and continuous deregulation of normal hepatocyte [Ca^2+^]_c_ could sensitize cells to signals that promote liver disease and/or HCC development. Importantly, HBx modulation of cytosolic Ca^2+^ is required for many of its effects in cells, indicating that HBx modulation of cytosolic Ca^2+^ is likely an upstream, initiating event that stimulates numerous downstream cellular functions [22, 26, 29]. Our studies provide insight into mechanisms that underlie HBx activities and HBV replication; factors that regulate these HBx activities could be novel targets for inhibiting HBx activities, HBV replication, and HBV pathogenesis.
